# Supplementary material for: Distribution pattern, molecular transmission networks, and phylodynamic of hepatitis C virus in China
Source: PLoS One. 2023 Dec 21;18(12):e0296053. doi: 10.1371/journal.pone.0296053 (PMC10734925; doi:10.1371/journal.pone.0296053)
Supplement: S1 Fig — The threshold that was selected is highlighted in red. (DOCX) [file pone.0296053.s001.docx]

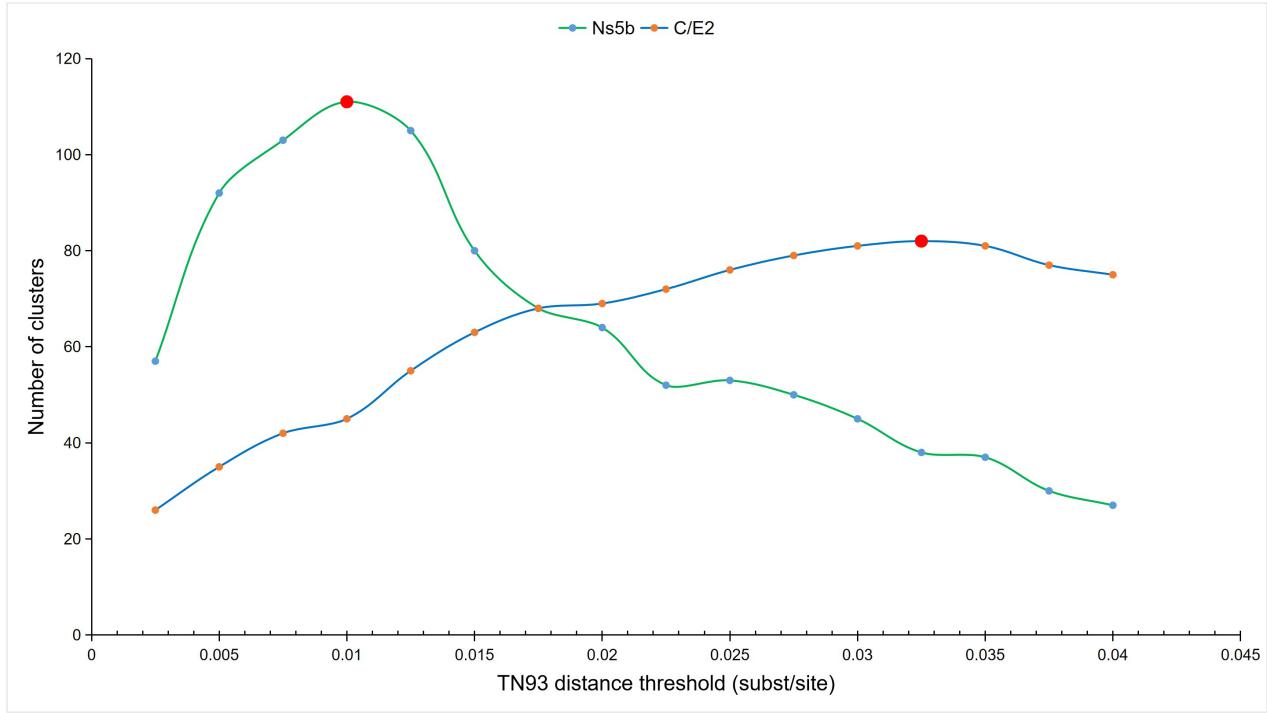


S1 Fig. Number of transmission clusters as a function of the TN93 distance. The threshold that was selected is highlighted in red.
