# Supplementary material for: Distribution pattern, molecular transmission networks, and phylodynamic of hepatitis C virus in China
Source: PLoS One. 2023 Dec 21;18(12):e0296053. doi: 10.1371/journal.pone.0296053 (PMC10734925; doi:10.1371/journal.pone.0296053)
Supplement: S2 Fig — 1,603 Ns5b and 865 C/E2 sequences from China were analyzed with HCV reference strains (NC_004102, D90208, AB047639, JN714194, JQ065709, HQ639936, DQ278894) as an out-group using Fasttree 2.1. (DOCX) [file pone.0296053.s002.docx]

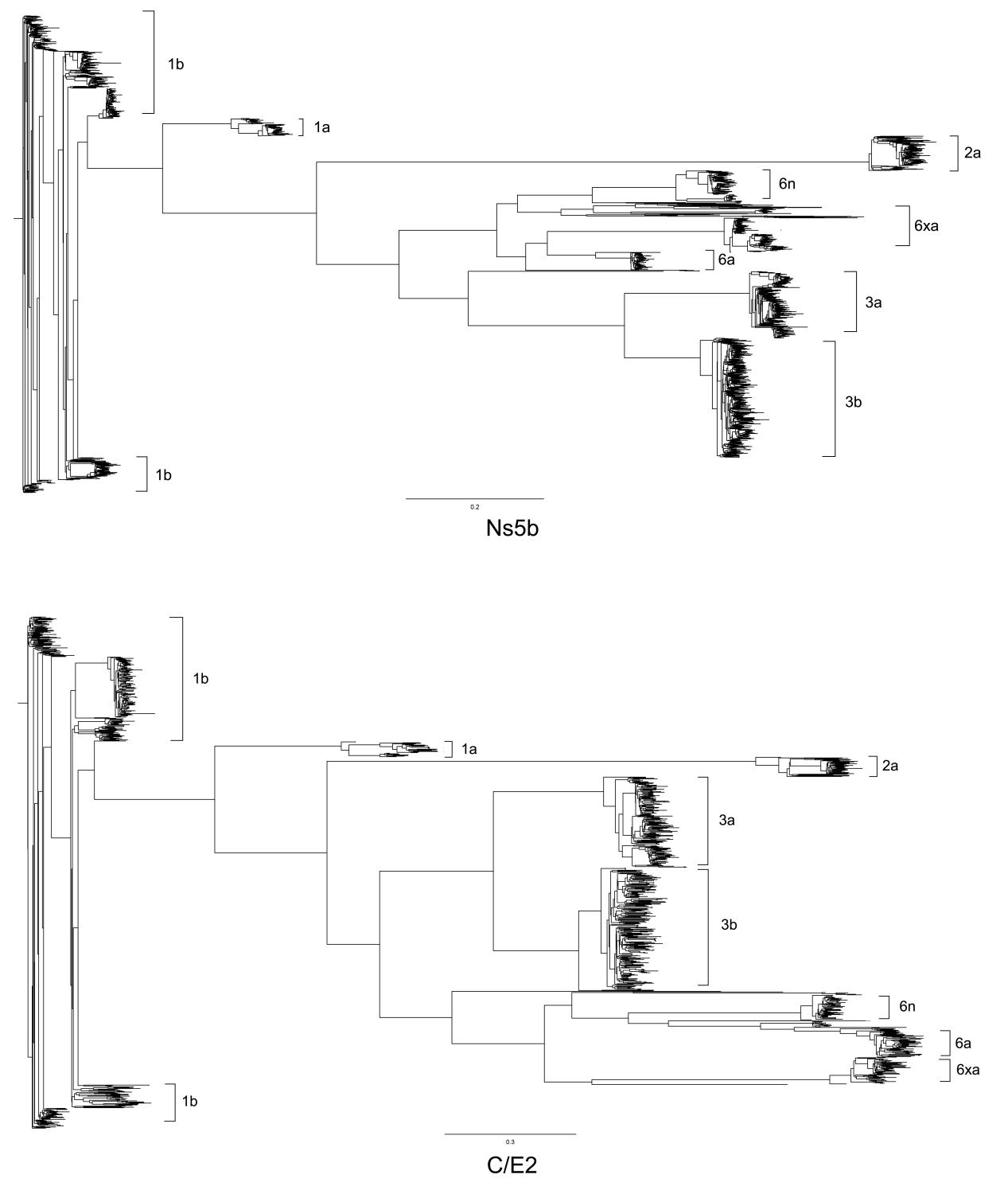


S2 Fig. The maximum likelihood (ML) phylogenetic tree based on *Ns5b* and *C/E2* gene. 1,603 Ns5b and 865 C/E2 sequences from China were analyzed with HCV reference strains (NC_004102, D90208, AB047639, JN714194, JQ065709, HQ639936, DQ278894) as an out-group using Fasttree 2.1.
