# Supplementary material for: Distribution pattern, molecular transmission networks, and phylodynamic of hepatitis C virus in China
Source: PLoS One. 2023 Dec 21;18(12):e0296053. doi: 10.1371/journal.pone.0296053 (PMC10734925; doi:10.1371/journal.pone.0296053)
Supplement: S3 Fig — TMRCA = Time to the Most Recent Common Ancestor. The solid line indicates the 95% highest posterior density [HPD] interval for TMRCA. (DOCX) [file pone.0296053.s003.docx]

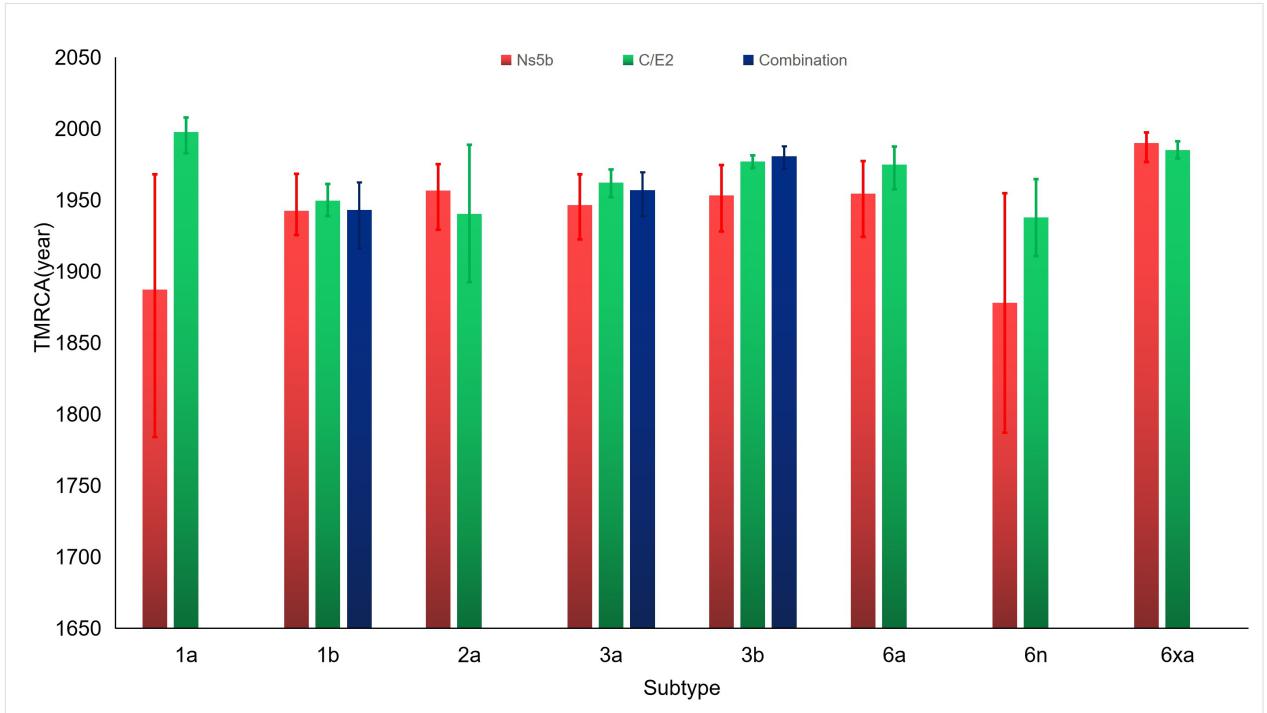


S3 Fig. The TMRCA of HCV in China.

TMRCA=Time to the Most Recent Common Ancestor.

The solid line indicates the 95% highest posterior density [HPD] interval for TMRCA.
