# Supplementary material for: Distribution pattern, molecular transmission networks, and phylodynamic of hepatitis C virus in China
Source: PLoS One. 2023 Dec 21;18(12):e0296053. doi: 10.1371/journal.pone.0296053 (PMC10734925; doi:10.1371/journal.pone.0296053)
Supplement: S4 Fig — The shaded portion is the 95% Bayesian credibility interval, and the solid line is the posterior median. (DOCX) [file pone.0296053.s004.docx]

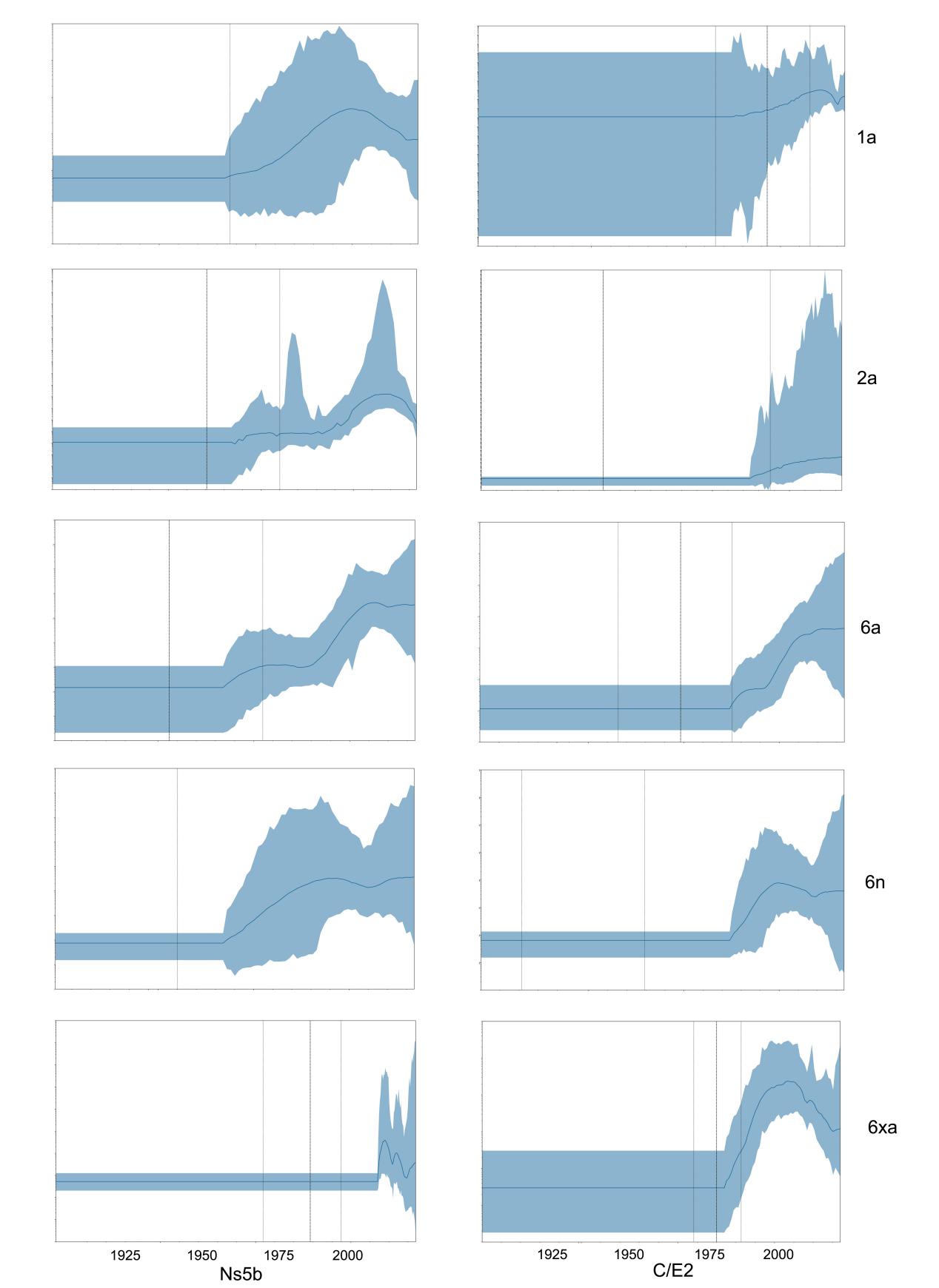


S4 Fig. The past population dynamics of HCV (1a, 2a, 6a, 6n, and 6xa) visualized using the Skygrid model. The shaded portion is the 95% Bayesian credibility interval, and the solid line is the posterior median.
