# Supplementary figures and images for: Distribution pattern, molecular transmission networks, and phylodynamic of hepatitis C virus in China
Source: PLoS One. 2023 Dec 21;18(12):e0296053. doi: 10.1371/journal.pone.0296053 (PMC10734925; doi:10.1371/journal.pone.0296053)

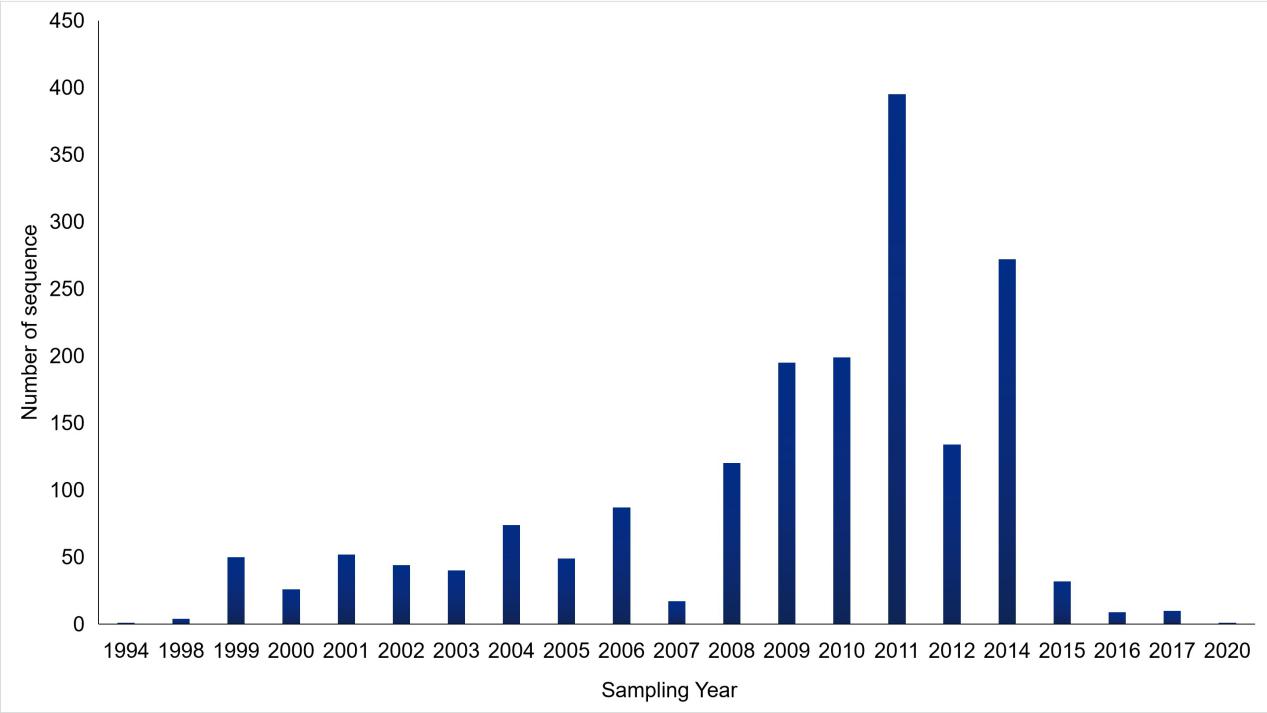


S5 Fig. The distribution of sampling year for HCV sequences in China.

Supplement: S5 Fig — (DOCX) [file pone.0296053.s005.docx]
