# Supplementary material for: Distribution pattern, molecular transmission networks, and phylodynamic of hepatitis C virus in China
Source: PLoS One. 2023 Dec 21;18(12):e0296053. doi: 10.1371/journal.pone.0296053 (PMC10734925; doi:10.1371/journal.pone.0296053)
Supplement: S1 Table — BHLN = Beijing HIV laboratory network; PWID = People who inject drugs; MSM = Men who have sex with men; NA = not available; LANL = Los Alamos National Laboratory. (DOCX) [file pone.0296053.s006.docx]

S1 Table. Descriptions of the participating cohort.

| Cohort | Description | Sampling period | Location | Subjects | HCV antibody-positive | HCV viraemia |
| --- | --- | --- | --- | --- | --- | --- |
| HIV TDR surveillance program in BHLN | A longitudinal cross-sectional study of HIV TDR in China. | 1999-2017 | Across China | 756 | 23 | 429 |
| Health examination population | A research project to study HCV prevalence in the general population who visited the health examination outpatient service of the Beijing CDC that used convenience sampling approaches. | 1999 | Across China | NA | 50 | 37 |
| MSM | A longitudinal cohort of MSM studying the incidence of HIV, HCV, and syphilis in MSM. | 2015-2021 | Across China | 4200 | 19 | 2 |
| LANL HCV sequence database sampled in China | A cross-sectional study to characterize all HCV sequences sampled in China with information on the province of origin and sample year. | 1994-2015 | Across China | 1343 | NA | 1343 |

TDR=Transmitted drug resistance;

BHLN=Beijing HIV laboratory network;

PWID=People who inject drugs;

MSM=Men who have sex with men;

NA=not available;

LANL=Los Alamos National Laboratory.
