# Supplementary material for: Distribution pattern, molecular transmission networks, and phylodynamic of hepatitis C virus in China
Source: PLoS One. 2023 Dec 21;18(12):e0296053. doi: 10.1371/journal.pone.0296053 (PMC10734925; doi:10.1371/journal.pone.0296053)
Supplement: S2 Table — North = Beijing, Hebei, Shanxi, Inner Mongolia, Northeast = Liaoning, Heilongjiang, East = Shanghai, Jiangsu, Zhejiang, Anhui, Jiangxi, Shandong, Central South = Henan, Hubei, Hunan, Guangdong,Guangxi, Hainan, Southwest = Chongqing, Sichuan, Guizhou, Yunnan, Northwest = Shannxi, Qinghai, Sinkiang; MSM = men who have sex with men, PWID = people who inject drugs; NA = not available; OR = odds ratio; aData are n (%); bUnivariable logistic regression analysis; cMultivariable logistic regression analysis; dData for n = 1552, eData for n = 1175, Other = 6e, 6g, 6l, 6w, and 6v. (DOCX) [file pone.0296053.s007.docx]

S2 Table. Demographic and clinical factors associated with clustering based on *Ns5b* gene.

|  |  |  | Univariable analysis^b^ | Multivariable analysis^c^ |
| --- | --- | --- | --- | --- |
|  | Number of sequences | Clustering frequency ^a^ | OR(95% CI) | OR(95% CI) |
| Region^d^ |  |  |  |  |
| North | 62 | 28(45.2) | Reference | Reference |
| Northeast | 8 | 1(12.5) | 0.17(0.01-1.06) | 0.2(0.01-1.25) |
| East | 307 | 77(25.1) | 0.41(0.23-0.72) | 0.37(0.19-0.71) |
| Central South | 357 | 93(26.1) | 0.43(0.25-0.75) | 0.48(0.26-0.90) |
| Southwest | 644 | 202(31.4) | 0.55(0.33-0.95) | 0.65(0.36-1.18) |
| Northwest | 174 | 114(65.5) | 2.31(1.28-4.19) | 2.83(1.5-5.36) |
| Population characteristic^e^ |  |  |  |  |
| Heterosexual | 4 | 1(25) | Reference |  |
| MSM | 2 | 0(0) | 0(0-0) |  |
| PWID | 905 | 345(38.1) | 1.85(0.24-37.44) |  |
| Former paid blood donor | 66 | 23(34.8) | 1.6(0.19-33.45) |  |
| General population | 197 | 61(31) | 1.35(0.17-27.5) |  |
| Volunteer blood donor | 1 | 1(100) | NA |  |
| Genotype and subtype |  |  |  |  |
| 1a | 58 | 24(41.4) | Reference | Reference |
| 1b | 466 | 218(46.8) | 1.25(0.72-2.19) | 1.19(0.66-2.19) |
| 2a | 115 | 16(13.9) | 0.23(0.11-0.48) | 0.23(0.1-0.52) |
| 3a | 225 | 60(26.7) | 0.52(0.28-0.95) | 0.32(0.17-0.61) |
| 3b | 404 | 90(22.3) | 0.41(0.23-0.73) | 0.33(0.18-0.6) |
| 6a | 126 | 44(34.9) | 0.76(0.4-1.45) | 0.88(0.45-1.74) |
| 6n | 107 | 41(38.3) | 0.88(0.46-1.7) | 0.88(0.45-1.73) |
| 6xa | 60 | 32(53.3) | 1.62(0.78-3.38) | 1.48(0.71-3.14) |
| Other | 42 | 5(11.9) | 0.19(0.059-0.52) | 0.27(0.08-0.77) |
| Period |  |  |  |  |
| 1994-2003 | 178 | 89(50) | Reference | Reference |
| 2004-2008 | 307 | 126(41) | 0.7(0.48-1.01) | 0.43(0.27-0.68) |
| 2009-2013 | 798 | 226(28.3) | 0.4(0.28-0.55) | 0.46(0.29-0.74) |
| 2014-2020 | 320 | 89(27.8) | 0.39(0.26-0.56) | 0.4(0.25-0.63) |

North=Beijing, Hebei, Shanxi, Inner Mongolia,

Northeast=Liaoning, Heilongjiang,

East=Shanghai, Jiangsu, Zhejiang, Anhui, Jiangxi, Shandong,

Central South=Henan, Hubei, Hunan, Guangdong,Guangxi, Hainan,

Southwest=Chongqing, Sichuan, Guizhou, Yunnan,

Northwest=Shannxi, Qinghai, Sinkiang;

MSM=men who have sex with men,

PWID=people who inject drugs;

NA=not available;

OR=odds ratio;

^a^Data are n (%) ;

^b^Univariable logistic regression analysis;

^c^Multivariable logistic regression analysis;

^d^Data for n=1552,

^e^Data for n=1175,

Other= 6e, 6g, 6l, 6w, and 6v.
