# Supplementary material for: Distribution pattern, molecular transmission networks, and phylodynamic of hepatitis C virus in China
Source: PLoS One. 2023 Dec 21;18(12):e0296053. doi: 10.1371/journal.pone.0296053 (PMC10734925; doi:10.1371/journal.pone.0296053)
Supplement: S3 Table — North = Beijing, Hebei, Shanxi, Inner Mongolia, Northeast = Liaoning, Heilongjiang, East = Shanghai, Jiangsu, Zhejiang, Anhui, Jiangxi, Shandong, Central South = Henan, Hubei, Hunan, Guangdong,Guangxi, Hainan, Southwest = Chongqing, Sichuan, Guizhou, Yunnan, Northwest = Shannxi, Qinghai, Sinkiang; MSM = men who have sex with men, PWID = people who inject drugs; NA = not available; OR = odds ratio; aData are n (%), bUnivariable logistic regression analysis, cMultivariable logistic regression analysis, dData for n = 849, eData for n = 846, Other = 6e, 6g, 6l, 6w, and 6v. (DOCX) [file pone.0296053.s008.docx]

S3 Table. Demographic and clinical factors associated with clustering based on *C/E2* gene.

|  |  |  | Univariable analysis^b^ | Multivariable analysis^c^ |
| --- | --- | --- | --- | --- |
|  | Number of sequences | Clustering frequency ^a^ | OR(95% CI) | OR(95% CI) |
| Region^d^ |  |  |  |  |
| North | 69 | 34(49.3) | Reference | Reference |
| Northeast | 11 | 1(9.1) | 0.1(0.01-0.58) | 0.1(0.005-0.57) |
| East | 250 | 31(12.4) | 0.15(0.08-0.27) | 0.17(0.09-0.34) |
| Central South | 51 | 9(17.6) | 0.22(0.09-0.51) | 0.23(0.09-0.54) |
| Southwest | 254 | 96(37.8) | 0.63(0.37-1.07) | 0.69(0.39-1.23) |
| Northwest | 214 | 140(65.4) | 1.95(1.12-3.38) | 1.88(1.07-3.28) |
| Population characteristic^e^ |  |  |  |  |
| Heterosexual | 4 | 1(25) | Reference |  |
| MSM | 1 | 0(0) | NA |  |
| PWID | 654 | 286(43.7) | 2.33(0.3-47.27) |  |
| Former paid blood donor | 73 | 13(17.8) | 0.65(0.08-13.71) |  |
| General population | 112 | 11(9.8) | 0.33(0.04-6.92) |  |
| Volunteer blood donor | 2 | 1(50) | 3(0.07-170.77) |  |
| Genotype and subtype |  |  |  |  |
| 1a | 24 | 8(33.3) | Reference | Reference |
| 1b | 290 | 118(40.7) | 1.37(0.58-3.48) |  |
| 2a | 32 | 0(0) | 0(0-0) |  |
| 3a | 155 | 60(38.7) | 1.24(0.52-3.28) |  |
| 3b | 211 | 86(40.8) | 1.37(0.58-3.52) |  |
| 6a | 45 | 11(24.4) | 0.65(0.22-1.96) |  |
| 6n | 60 | 13(21.7) | 0.55(0.19-1.62) |  |
| 6xa | 43 | 21(48.8) | 1.91(0.69-5.59) |  |
| Other | 5 | 0(16.7) | 0(0-0) |  |
| Period |  |  |  |  |
| 1994-2003 | 103 | 63(61.2) | Reference | Reference |
| 2004-2008 | 222 | 109(49.1) | 0.61(0.38-0.98) | 0.54(0.36-1.06) |
| 2009-2013 | 491 | 123(25.1) | 0.21(0.14-0.33) | 0.55(0.31-0.96) |
| 2014-2020 | 49 | 22(44.9) | 0.52(0.26-1.03) | 0.67(0.31-1.43) |

North=Beijing, Hebei, Shanxi, Inner Mongolia,

Northeast=Liaoning, Heilongjiang,

East=Shanghai, Jiangsu, Zhejiang, Anhui, Jiangxi, Shandong,

Central South=Henan, Hubei, Hunan, Guangdong,Guangxi, Hainan,

Southwest=Chongqing, Sichuan, Guizhou, Yunnan,

Northwest=Shannxi, Qinghai, Sinkiang;

MSM=men who have sex with men,

PWID=people who inject drugs;

NA=not available;

OR=odds ratio;

^a^Data are n (%) ,

^b^Univariable logistic regression analysis,

^c^Multivariable logistic regression analysis,

^d^Data for n=849,

^e^Data for n=846,

Other= 6e, 6g, 6l, 6w, and 6v.
