# Supplementary material for: Distribution pattern, molecular transmission networks, and phylodynamic of hepatitis C virus in China
Source: PLoS One. 2023 Dec 21;18(12):e0296053. doi: 10.1371/journal.pone.0296053 (PMC10734925; doi:10.1371/journal.pone.0296053)
Supplement: S4 Table — TMRCA = Time to the Most Recent Common Ancestor. aData are TMRCA (the 95% highest posterior density [HPD] interval). (DOCX) [file pone.0296053.s009.docx]

S4 Table. The TMRCA of HCV in China inferred from original dataset.

|  | *Ns5b* |  | *C/E2* |  |
| --- | --- | --- | --- | --- |
| Subtype | Numbers | TMRCAa | Numbers | TMRCAa |
| 1a | 58 | 1887.5(1784-1968.3) | 25 | 1890.3(1149.5-1991.7) |
| 1b | 466 | 1993(108.9 B.C.-1185.6) | 292 | 1663.1(1500.6-1789.3) |
| 2a | 115 | 1775(520.6 B.C.-1217.9) | 33 | 1962.2(1952.2-1971.5) |
| 3a | 224 | 1774(1920.1-1972.8) | 155 | 1676(1454.6-1835.2) |
| 3b | 404 | 1973(1512.1 B.C.-367.3) | 213 | 1809.5(1638.8-1930.2) |
| 6a | 126 | 1968(2992.9 B.C.-898.2) | 45 | 1937(1876.3-1975.9) |
| 6n | 107 | 1956(1865.8-1980.2) | 60 | 1495.5(989.8-1779.9) |
| 6xa | 60 | 735(1536.1-1918.6) | 43 | 1713.2(827.1-1983.6) |

TMRCA=Time to the Most Recent Common Ancestor.

aData are TMRCA (the 95% highest posterior density [HPD] interval).
