# Supplementary material for: Distribution pattern, molecular transmission networks, and phylodynamic of hepatitis C virus in China
Source: PLoS One. 2023 Dec 21;18(12):e0296053. doi: 10.1371/journal.pone.0296053 (PMC10734925; doi:10.1371/journal.pone.0296053)
Supplement: S1 File — (DOCX) [file pone.0296053.s010.docx]

S1 File.

The protocol for Bayesian estimation of past population dynamics using the Skygrid coalescent model

1.We construct 19 data set containing the ns5b gene sequence for HCV subtype 1a, 1b, 2a, 3a, 3b, 6a, 6n, 6xa; C/E2 gene sequence for subtype 1a, 1b, 2a, 3a, 3b, 6a, 6n, 6xa; combined sequence for subtype 1b, 3a, and 3b.

2.Sequences were aligned using the BioEdit tool and the alignment was manually corrected according to the encoded reading frame. The sequences labels are all set in the same format with sample ID separated by the prefix "_" and contain the sampling year, which simplifies the various steps in this analysis.

3."Nonclock" phylogenetic trees were constructed using the Kimura 2-parameter model, with 1,000 bootstrap replicates, using the Mega 11 software.

4. Open a file chooser to select the "nonclock" phylogenetic tree in the TempEst. Click on the Sample Dates tab at the top of the screen, then click on the Parse Dates button to extract the sampling year for each sequence in the alignment.

5. In the Root-to-tip panel, we did the regression analysis of genetic divergence from the root of the tree against year of sampling. The sequences whose sampling year is incongruent with genetic divergence were excluded for Bayesian analysis.

6. We import our 19 FASTA files into BEAUti, a graphical user interface designed for creating XML files for input into BEAST;

7. Start the BEAUti application and drag-and-drop each FASTA file onto the Partitions panel.

8. Move on to the Tips panel, obtain the sampling year of the sequences in the same manner by selecting Parse Dates;

9. Move on to the Sites panel, select the coding ("196_CDS") partition and choose "HKY" from the first drop-down menu. Leave Base frequencies unchanged, select "Gamma" from the Site Heterogeneity Model drop-down menu, and keep the default of our gamma categories; Select '3 partitions: positions 1, 2, 3' from the Partition into codon positions drop-down menu, and ensure that all three check boxes are ticked;

10. Move on to the Clock panel, select the 'Uncorrelated relexed clock' from the Clock Type dropdown menu; In the Trees tab, click on the Tree Prior drop-down menu and select 'Coalescent: Bayesian SkyGrid'. We keep the default option of a Random starting tree to start the inference process.

11. Move on to the MCMC panel, put 100 million in the Length of chain field. Click on Generate BEAST file in the bottom of right-hand corner to save the XML file containing the provided information.

12. Start BEAST and select the XML file that was generated using BEAUti. Click Run to start the analysis;

13. Launch Tracer, click on Open in the menu bar, and select the .log files from BEAST runs. Select the Skygrid Reconstruction option from the Analysis menu.
