# Supplementary material for: Distribution pattern, molecular transmission networks, and phylodynamic of hepatitis C virus in China
Source: PLoS One. 2023 Dec 21;18(12):e0296053. doi: 10.1371/journal.pone.0296053 (PMC10734925; doi:10.1371/journal.pone.0296053)
Supplement: S2 File — (DOCX) [file pone.0296053.s011.docx]

S2 File.

Accession numbers

AF040777,EU081314-EU081328,EU081442-EU081508,EU158186,FJ462948-FJ463005,FJ463030,GQ285027-GQ285071,HQ318837,HQ318848,HQ318849,HQ318863-HQ318927,JN870282,JQ065709,JQ303356-JQ303643,JQ797684-JQ797691,JX960977-JX961173,KC441467-KC441486,KC878883,KC878884,KC878894,KC878895,KC878897-KC879030,KF292122-KF292179,KM284805,KM284809,KM284812,KM284813,KM284822,KM284823,KM284830,KM284831,KM284832,KM284835,KM284841,KM284842,KM284845,KM284848,KM284849,KM284851,KM284855,KM284857-KM284859,KM284862,KM284863,KM284867,KM284878,KM284882,KM284891,KM284893,KM284895,KM284898-KM284900,KM284902,KM284904,KM284908,KM284910,KM284911,KM284915,KM284918,KM284920,KM284921,KM284927,KM284928,KM284930,KM284932,KM284934,KM284935,KM284936,KM284944,KM284948,KM284949,KM284956,KM284962,KM284964,KM284965,KM284969,KM284972,KM284978-KM285124,KT735659-KT735898,KY120328-KY120332,KY704892-KY704918,MG878999,MG879000,MK139015-MK139022,MK327981,MK327984-MK328023,MK328026;

AF040777,EU119968-EU119983,EU158186,HQ318826-HQ318856,JN870282,JQ065709,JQ303352-JQ303509,KC878878-KC878937,KM284803-KM284972,KM284974,KM284976,KY120328-KY120329-KY120332,KY704892-KY704918,MK139015-MK139022,MK327986;

OR096896 - OR097294, OR097295 - OR097690.
