# Supplementary material for: Distribution pattern, molecular transmission networks, and phylodynamic of hepatitis C virus in China
Source: PLoS One. 2023 Dec 21;18(12):e0296053. doi: 10.1371/journal.pone.0296053 (PMC10734925; doi:10.1371/journal.pone.0296053)
Supplement: Attachment — Submitted filename: HCV plos second comment.docx [file pone.0296053.s013.docx]

PONE-D-22-30314R1

Distribution pattern, molecular transmission networks, and phylodynamic of hepatitis C virus in China

Dear Editor and Reviewers:

We are very grateful to you for giving us an opportunity to revise our manuscript. We really appreciate you very much for your positive and constructive comments and suggestions on our manuscript. We have studied Poon' comments carefully and tried our best to revise our manuscript according to the comments. The following are the responses and revisions we have made in response to the Poon' questions and suggestions point-by-point.

Thanks again to the hard work of the editor and reviewers.

Dear Dr. Lu,

Thank you for submitting your manuscript to PLOS ONE. After careful consideration, we feel that it has merit but does not fully meet PLOS ONE’s publication criteria as it currently stands. Therefore, we invite you to submit a revised version of the manuscript that addresses the points raised during the review process.

Please address the concerns raised by Reviewer #1 in the revised manuscript prior to its acceptance.

Reviewer #1: The authors were diligent in making revisions to their manuscript in response to reviews.

Response: Thank you for your positive comments. I learn a lot from your comments. Thanks.

* "The sequences whose sampling year is incongruent with genetic divergence were excluded for Bayesian analysis."

I apologize for making myself clearer in my previous review - my intention was to recommend that the authors should use TempEst to evaluate each data set for evidence of a molecular clock, and then to *discard the entire alignment for a given HCV subtype* if it did not meet this criterion. Individually discarding a substantial number of sequences based on a clock-based criterion is dangerous, because one can reshape a data set that has no temporal signal into one that strongly supports a clock. Rather, I think this treatment is associated with identifying sequences with incorrect dates, or from external evidence of systematic and substantial sequencing errors, for example.

As a compromise:

1. Please provide your exact quantitative criteria for filtering sequences at this step.

Response:

I agree with your opinion concerning discarding a substantial number of sequences based on clock-based criterion.

Our study invariably falls within the caveat of sampling bias.

We acknowledge this in the limitations section.

In HIV molecular epidemiology studies, researchers always need to discard some sequences either to reduce the computational load or to avoid potential bias that may arise from over-sampling a particular location.

In discarding sequences, we referred to the article by Andrew Rambaut,et al. entitled “Exploring the temporal structure of heterochronous sequences using TempEst (formerly Path-O-Gen).” published in Virus Evol 2016;2(1):vew007.

The quantitative criterion for filtering sequences is their positions in the root-to-tip plot.

Outliers were carefully investigated and discarded if they were suspected of having quality problems.

In total, we discarded 152(6.5%) sequences from the original datasets which consist of 2342 sequences.

The problems for these outliers sequences were 1)low sequencing quality, 2)errors in sequence assembly, 3) alignment error in part of the sequence.

The majority of the 152(88.2%) sequences were retrieved from GenBank.

The TMRCA of the original datasets has a larger interval and lower ESS value, suggesting that including sequences with problems could bias the inference.

2. I am somewhat concerned that the results were quite sensitive to filtering sequences, i.e., estimates of TMRCA. The most transparent thing to do here would be to present both sets of results: (1) the original results without filtering sequences, and (2) results having filtered sequences under clearly reported criteria. The original results can be moved to Supplementary Materials to minimize revisions to the main text.

Response:

We provided the original results without filtering sequences in the Supplementary Materials.

* "For each dataset, three independent Markov chain Monte Carlo (MCMC) chains were run for 100 million generations with states sampled every 10,000 generations. Log files were combined using Logcombiner to ensure sufficient convergence [...]"

More miscommunication here. The comment in my previous review was: "The results would also be more convincing if the authors had run replicate chains for each dataset to demonstrate convergence." I should have clarified that the replicate chain samples should be compared for evidence of convergence, *e.g.* overlapping posterior traces, before combining samples. It does seem that the authors did something like this, however, given the following line: "The convergence of MCMC chains was checked using Tracer (version 1.7.2)" If this is correct, then please provide the specific criteria you used to determine convergence - even visual assessment is okay. (On reflection, I suppose that one might demonstrate convergence if combining chains increases ESS.)

Response:

The biggest difficulty we faced was the inclusion of too many datasets(8 NS5B+8 CE2+3 combination) in our analysis.

Moreover, we did not down-sample sequences to maintain the integrity of the original data.

Therefore, the computational load was large.

We ran multiple MCMC and combined the log files to increase ESS.

Fortunately, of the 19 datasets, only six have ESS value less than 200 in Bayesian inference.

We only had to run multiple for these six datasets.

Before we combine these results, we compare the evidence of convergence, e.g. ,statistics such as joint, prior, and likelihood.

I beg your pardon for the meaning of “overlapping posterior traces.”

We referred to at least nine classic papers on Bayesian evolutionary analysis, and none of them provided direct answer.

Would you kindly recommend some manuscripts concerning “overlapping posterior traces” .

Your kindness and help are greatly appreciated.

1.Nuno R Faria,et al.HIV epidemiology. The early spread and epidemic ignition of HIV-1 in human populations.Science 2014;346(6205):56-61.

2.Claudia Palladino,et al.Epidemic history of hepatitis C virus genotypes and subtypes in Portugal.Sci Rep 2018;8(1):12266.

3.Medhat K Shier ,et al.Molecular characterization and epidemic history of hepatitis C virus using core sequences of isolates from Central Province, Saudi Arabia.PLoS One

2017;12(9):e0184163.

4.Oliver G. Pybus,Eleanor Barnes,Rachel Taggart,et al.Genetic History of Hepatitis C Virus in East Asia.J Virol 2009; 83,1071-82.

5.Anna L McNaughton, Iain Dugald Cameron,Elizabeth B Wignall-Fleming,et al.2015. Spatiotemporal reconstruction of the introduction of hepatitis C virus into Scotland and its subsequent regional transmission. J Virol 2015; 89:11223–11232.

6.K Hoshino ,et al.Phylogenetic and phylodynamic analyses of hepatitis C virus subtype 1a in Okinawa, Japan.J Viral Hepat 2018;25(8):976-985.

7.S Zhou,E Cella,W Zhou,et al. Population dynamics of hepatitis C virus subtypes in injecting drug users on methadone maintenance treatment in China associated with economic and health reform. J Viral Hepat 2017; 551-560.

8.Anna L McNaughton, Iain Dugald Cameron,Elizabeth B Wignall-Fleming,et al.2015. Spatiotemporal reconstruction of the introduction of hepatitis C virus into Scotland and its subsequent regional transmission. J Virol 2015; 89:11223–11232.

9.Peter V Markov,et al.Colonial history and contemporary transmission shape the genetic diversity of hepatitis C virus genotype 2 in Amsterdam.J Virol 2012 ;86(14):7677-87.

* the Open Science Framework URL does not work as written. Please use <https://doi.org/10.17605/OSF.IO/NKD8Y.>

Response: We used it.

We acknowledge that we have not encompass all of your comments.

We hope that these responses are acceptable.

Thank you very much.

Yours sincerely

Hongyan Lu.
